# Sexual Dimorphism in *Anthonomus santacruzi* (Coleoptera: Curculionidae): a Biological Control Agent of *Solanum mauritianum* Scopoli (Solanaceae)

**DOI:** 10.1007/s13744-020-00795-6

**Published:** 2020-07-16

**Authors:** Archbold Sasa, Rafał Gosik, Ed T. F. Witkowski, Marcus J. Byrne, Miłosz A. Mazur

**Affiliations:** 1 School of Animal, Plant & Environmental Sciences, Univ. of the Witwatersrand, Johannesburg, South Africa; 2 Dept. of Zoology and Nature Protection, Maria Curie–Skłodowska University, Lublin, Poland; 3 DST–NRF Centre of Excellence for Invasion Biology, School of Animal, Plant & Environmental Sciences, Univ. of the Witwatersrand, Johannesburg, South Africa; 4 Institute of Biology, Univ. of Opole, Oleska 22, 45-052 Opole, Poland

**Keywords:** Biocontrol, invasive plant, morphometry, sex determination, sexual dimorphism, South Africa

## Abstract

There is evident variation in body size amongst *Anthonomus santacruzi* Hustache, 1924, weevils. The aims of this study were to assess if the variation in body size in *A*. *santacruzi* weevils is a result of sexual dimorphism and what features can be used to distinguish males from females. The weevils were collected from field sites in Mpumalanga, South Africa, where they were introduced as biocontrol agents of *Solanum mauritianum* Scopoli. Body structures and the presence/absence of the tergal notch was examined under an optical stereomicroscope and SEM to assess differences between sexes. The morphometric analysis of the body structures included rostrum length (base–apex and antennal insertion–apex), elytral length and width, pronotum length and width, first tarsus length, first tibia length, funiculus length and total body length. Rostrum length, elytra length and width and total body length were significantly larger in females than in males. A tergal notch in the 8th abdominal tergite was present in males and absent in females. The body structure; of rostrum length, elytra length and width and total body length overlapped between sexes in some specimens. The abdominal tergal notch was found to be the most useful body structure to distinguish males from females in *A*. *santacruzi*.

**Electronic supplementary material:**

The online version of this article (10.1007/s13744-020-00795-6) contains supplementary material, which is available to authorized users.

## Introduction

*Anthonomus* Germar, 1817 (Coleoptera: Curculionidae) is a weevil genus containing more than 500 species, distributed predominantly in the Neotropic (over 400 species) and in the Palearctic (73 species) regions, but also in Indomalaya (9 species), the Afrotropics (3 species) and in Australasia (2 species) (Bená & Vanin 2013; Gosik *et al* 2017). They develop inside buds and fruits, and the adults are known to feed on buds, flowers, fruits and young foliage of several cultivated and wild plants, resulting in premature abscission (Rodríguez Leyva *et al* 2007; Speranza *et al* 2014). Therefore, the genus includes many species of economic importance such as *Anthonomus grandis* Boheman, 1843, *A. eugenii* Cano, 1894 and *A. rubi* (Herbst, 1795), which are regarded as serious pests (Smreczyński 1972, Ramalho & Jesus 1988, Speranza *et al* 2014). On the other hand, species such as *A*. *santacruzi* Hustache, 1924, *A*. *monostigma* Champion, 1903, *A*. *morticinus* Clark, 1998 and *A*. *sisymbrii* Hustache, 1939 are used as biological control agents or being considered as potential biocontrol agents of invasive alien plants in USA, South Africa and New Zealand (King *et al* 2011; Chacón Madrigal *et al* 2012; Hakizimana and Olckers 2013; English & Olckers 2018; Mkhize & Olckers 2019). According to Clark & Burke (1996), over 30 species of *Anthonomus* are associated with Solanaceous plants.

**Fig 1:**
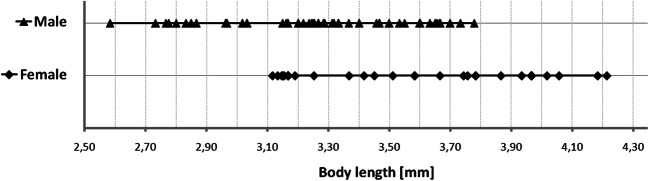
Range in total length between male and female *Anthonomus santacruzi* Hustache, 1924.

*Anthonomus santacruzi* (flowerbud weevil) (Fig 1) is native to South America (southern Brazil, northern Argentina and Paraguay) and feeds mainly on the flowers and flower buds of *Solanum mauritianum* (Olckers 2011). The habitat, food and ecological preferences of *A*. *santacruzi* adults were described by Clark & Burke (1996), Olckers (2003) and Mkhize and Olckers (2019), while the morphology of the immature stages was described by Gosik *et al* (2017). The weevil was released in South Africa in 2008 for the biological control of *S. mauritianum* (Olckers 2011; Fowler 2014; English & Olckers 2018) and has established, but was subsequently rejected in New Zealand in 2013 (for non-target attacks on *Solanum aviculare* in quarantine (Hakizimana and Olckers 2013)*. S. mauritianum* is a major declared weed, predominantly in the eastern higher (summer) rainfall regions of South Africa, negatively impacting agricultural lands, plantation forestry, riverine habitats and conservation areas (see Cowie *et al* 2018 for a global review). It has a high reproductive rate in terms of seed production, and dense, relatively long-lived soil seed banks, resulting in a proliferation of seedlings when released from canopy shading (Witkowski & Garner 2008). Furthermore, mechanical clearing by cutting the stem is largely ineffective due to basal resprouting, unless the stem is cut low and the cut stump immediately treated with the appropriate herbicide (Witkowski and Garner 2008). Reducing its reproductive output through releasing appropriate biocontrol agents should be a priority. Furthermore, when releasing the agents onto selected *S. mauritianum* populations, the optimal ratio of males to females should be utilized to maximize population development and growth and hence achieve the intended control effects. The use of *A*. *santacruzi* as a weed biological control agent in South Africa justifies research into its biology and ecology.

Separating male and female *A*. *santacruzi* pupae using morphological characters other than the shape of the gonothecae is difficult (Gosik *et al* 2017). It was found, that female pupae (average body length = 2.76 mm) were larger than male pupae (average body length = 2.52 mm). However, the phenotypic variation in body size, so evident in *A*. *santacruzi* weevil populations, has still not been clearly verified to assess whether it is due to sexual dimorphism or only depends on variation between individual specimens, that might be related to external environmental factors such as food quality (Foelker & Hofstetter 2014; Łukowski *et al* 2015). Further, recognizing the differences between sexes using external morphology is unknown for this species.

According to Dinuă *et al* (2009), in many cases, the two sexual forms in insects cannot be separated without analysing the genital organs. However, sexes in some species can be distinguished using secondary characters such as the body size and proportion of particular parts, the form of the antennae and oral ornaments, sculpture of the cuticle, body colour and more developed appendices in one of the sexes, as well as functional sexual characters (e.g. a large number of olfactory sensors and more developed antennae on males, or a bigger abdomen on females).

Body size is a key biological trait which is frequently used to assess fitness. Apart from sexual dimorphism, variation in body size may also stem from genetic heritability and environmental factors and have strong effects on reproduction, dispersal ability and intraspecific competition (Foelker & Hofstetter 2014). Environmental factors such as temperature, habitat and resource food quality have been found to cause phenotypic variation in insect body sizes (Foelker & Hofstetter 2014).

Since there is apparent variation in body size in *A*. *santacruzi* populations, the ability to distinguish the sexes may be important in post-release evaluation of the weevil in biological control studies. For example, in experiments which require the sex of the experimental or sampled weevils to be known (Sappington & Spurgeon 2000). The precise estimation of the sex ratio is an important issue in ecological and biological control programs (Tabadkani *et al* 2012). Distinguishing sexes may also help not only in explaining the relative differences in body sizes in *A*. *santacruzi* but also in complementing a general gap in knowledge about the external sexual dimorphism of insects in general. Therefore, the aim of this study is to explore morphological differences between *A*. *santacruzi* males and females and to provide a useful tool for rapid differentiation between the sexes, both during laboratory procedures and field work.

## Material and Methods

A total of 97 weevils were collected (by hand) from randomly selected *S. mauritianum* plants in the field in the Sabie River catchment, Mpumalanga, South Africa (GPS S25°03′45.0″, E30°54′25.2″, altitude 766 m a.s.l.) in November 2017 by A. Sasa and were immediately fixed in 75% ethanol.

To find morphological characters important for sex determination of *A. santacruzi* we assessed some of the features studied by Smreczyński (1972) on *A. varians* (Paykull, 1792), on *A. pomorum* by Duan *et al* (1999), by Sappington & Spurgeon (2000) on *A. grandis* and on the subfamily Anthonominae by Kovarik (1983). We also assessed some characters used for sex differentiation in other weevils from different subfamilies, e.g. *Hylobius warreni* Wood, 1957 (Öhrn *et al* 2008), as well as other potentially sexually dimorphic features such as the shape of pronotum and length of the funicle.

Nine morphometric variables, namely (1) total length of the rostrum (from base to apex); (2) metarostrum (using the distance between the base of the rostrum and the insertion of the scape); (3) the length of the pronotum (measured along the medial line); (4) width of pronotum (measured at the widest part); (5) length of elytra (measured along elytral suture); (6) width of elytra (measured in the widest place); (7) length of the first tarsus (measured without the tarsal claw); (8) length of the first tibia; and (9) length of the funicle, were examined and measured on all 97 weevil bodies under an optical stereomicroscope (Olympus SZ11). Measurements were made by using a calibrated ocular micrometre at × 60 magnification.

We also examined selected non-morphometric characters such as the sculpturing on the rostrum and pronotum, shape and sculpturing of the elytra and shape of abdominal sternites to check their usefulness in sex determination.

The presence/absence of the notch in the ventral portion of the 8th abdominal sternite of the male, as described by Agee (1964) and Sappington & Spurgeon (2000), as a sex distinguishing structure on *A. grandis* was also observed, as well as the differences of the 7th and 8th abdominal segments recorded as being sex related in *A. pomorum* (Duan *et al* 1999).

Finally, all studied specimens were dissected to ascertain their sex and stored in separate, numbered tubes. Body length was calculated as the sum of rostrum, head, pronotum and elytra length. The differences in body characters between males and females were analysed using *t* tests and regarded as significantly different at *P* < 0.05.

Contrasting male and female photos, illustrating various body structures (whole body, rostrum at base–apex, rostrum at base–antenna (metarostrum), differences in the 4th and 5th abdominal segments, tergal notch, antenna and olfactory sensillae), were taken with an Olympus BX63 microscope and processed by Olympus cellSens Dimension software. The specimens selected for pictures using SEM (scanning electron microscopy) were at first dried in absolute ethyl alcohol (99.8%), rinsed in acetone and then gold-plated. A TESCAN Vega 3 SEM was used for the examination of selected structures. All these specimens were deposited in the collection of the Department of Zoology and Nature Protection, Maria Curie–Skłodowska University (Lublin, Poland).

## Results

### Morphological comparisons between sexes

#### Characters with significant sex-related differences

##### Total body length

Female body length was significantly greater (mean = 3.581 mm, *n* = 32) than male body length (mean 3.254 mm, *n* = 65) in all specimens (*n* = 97) (*t*_(95)_ = 4.911, *P* < 0.001) (Fig. 1, Table 1).

**Table 1:** *Anthonomus santacruzi* Hustache, 1924, sex determination character measurements between all male and female specimens (mean ± SD, *n* = 97).

Character	Females (mm) (*n* = 32)	Female range (mm)	Males (mm) (*n* = 65)	Male range (mm)	*P* value
Rostrum length (base–apex)	0.928 ± 0.09	0.75–1.08	0.809 ± 0.065	0.7–0.933	*< 0.001*
Metarostrum (base-antenna)	0.455 ± 0.062	0.25–0.55	0.363 ± 0.049	0.25–0.477	*< 0.001*
Pronotum length	0.489 ± 0.047	0.417–0.583	0.485 ± 0.044	0.383–0.6	0.668
Pronotum width	0.689 ± 0.069	0.55–0.833	0.674 ± 0.065	0.5–0.83	0.284
Elytra length	1.511 ± 0.173	1.167–1.767	1.422 ± 0.178	0.967–1.75	*0.022*
Elytra width	1.111 ± 0.132	0.833–1.433	1.043 ± 0.097	0.8–1.217	*0.005*
First tarsus length	0.299 ± 0.055	0.2–0.383	0.294 ± 0.056	0.2–0.483	0.658
First tibia length	0.526 ± 0.1	0.33–0.8	0.529 ± 0.067	0.367–0.667	0.841
Funiculus length	0.492 ± 0.057	0.383–0.633	0.479 ± 0.053	0.35–0.6	0.279
Total body length	3.581 ± 0.365	3.117–4.213	3.254 ± 0.277	2.583–3.778	*<0.001*
Notch	Absent	–	Present	–	–

*P values in italic indicate significant differences between male and female body characters at *P* < 0.05 (*t* test).

##### Elytra shape and morphometrics

**Fig 2:**
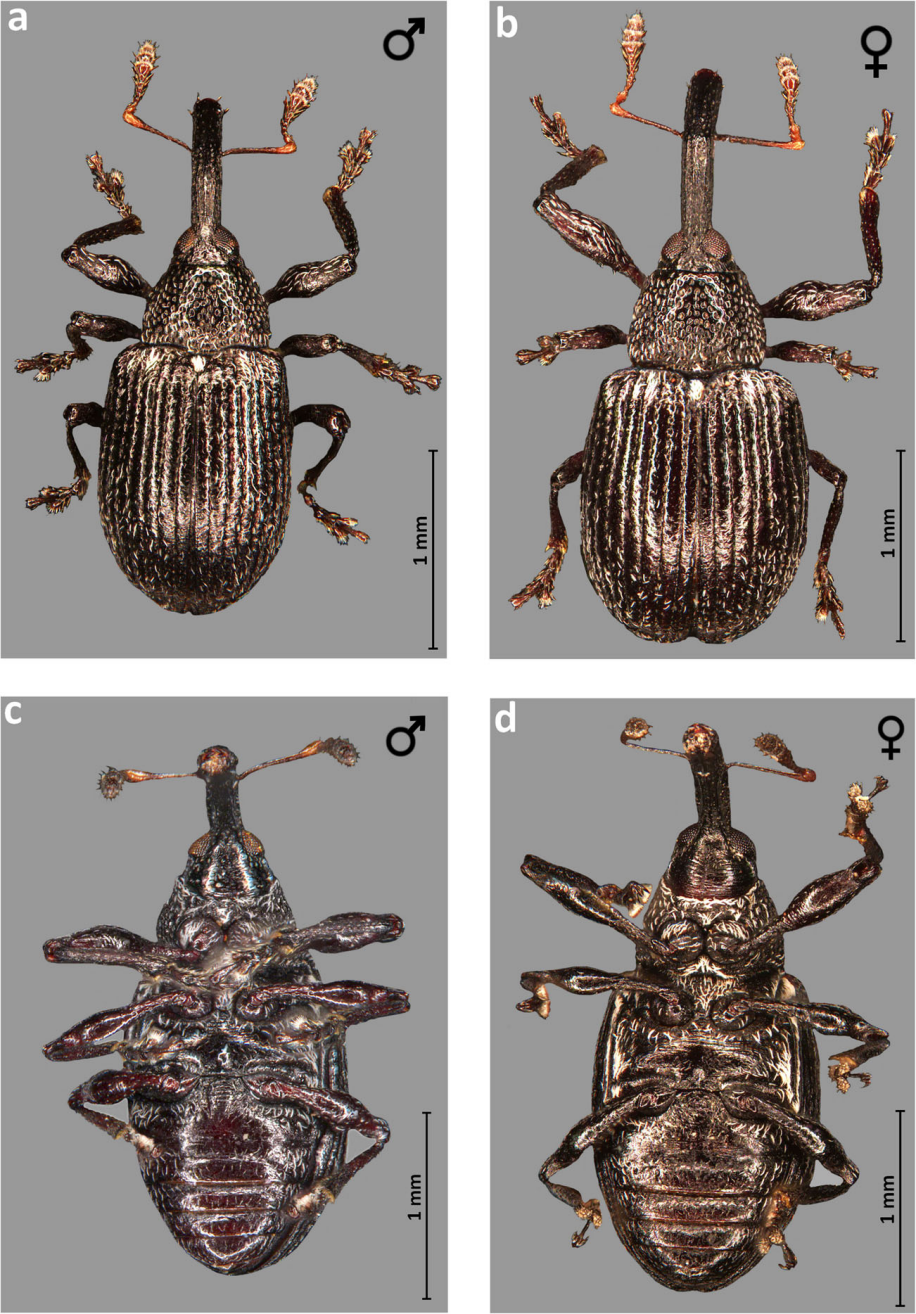
*Anthonomus santacruzi* Hustache, 1924, habitus: **a** dorsal view of male; **b** dorsal view of female; **c** ventral view of male; **d** ventral view of female (**a**–**d** optical microscope pictures).

**Fig 3:**
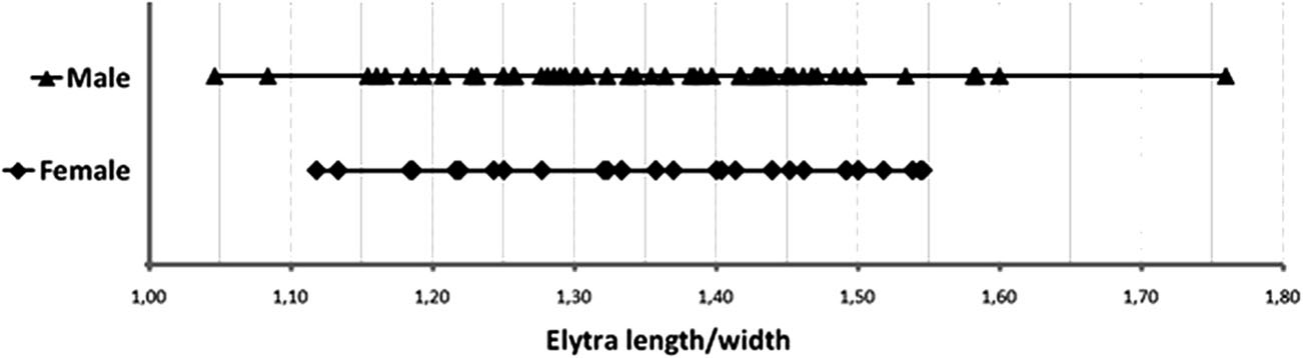
Length/width ratio of elytra in male and female *Anthonomus santacruzi* Hustache, 1924.

**Fig 4:**
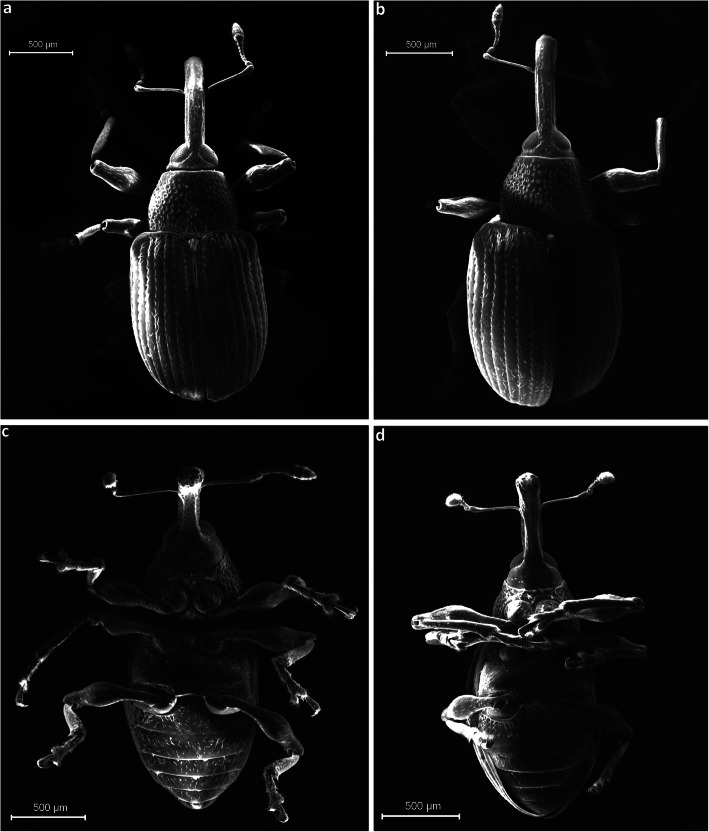
*Anthonomus santacruzi* Hustache, 1924, habitus: **a** dorsal view of male; **b** dorsal view of female; **c** ventral view of male; **d** ventral view of female (**a**–**d** SEM pictures).

Elytra were both significantly longer (mean = 1.511 mm) (*t*_(95)_ = 2.382, *P* = 0.022) and wider (1.111 mm) (*t*_(95)_ = 2.903, *P* = 0.005) in females than in males (length = 1.422 mm and width = 1.043) (Table 1), (Figs 2a–d) and were shaped somewhat differently, almost parallel on males vs. slightly enlarged on the distal part on females. The shape of elytra in males is variable, could be either almost subquadrate or distinctly elongate (Figs 2a–d, 3 and 4a–d).

##### Rostrum morphology

**Fig 5:**
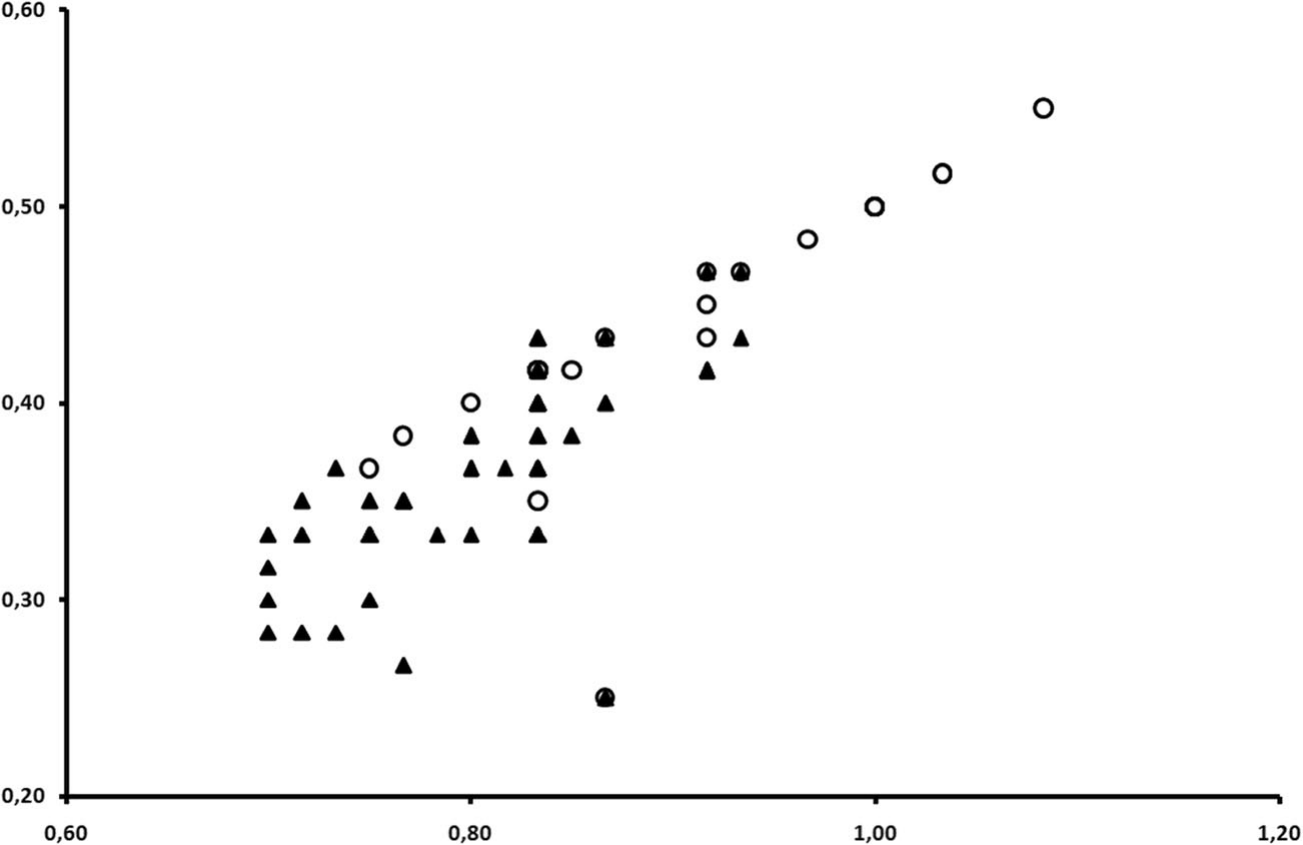
The relationship between the total length of the rostrum and length of the metarostrum, between male and female (X-axis - length of rostrum, Y-axis - length of metarostrum; black triangle - male, white circle - female) *Anthonomus santacruzi* Hustache, 1924.

**Fig 6:**
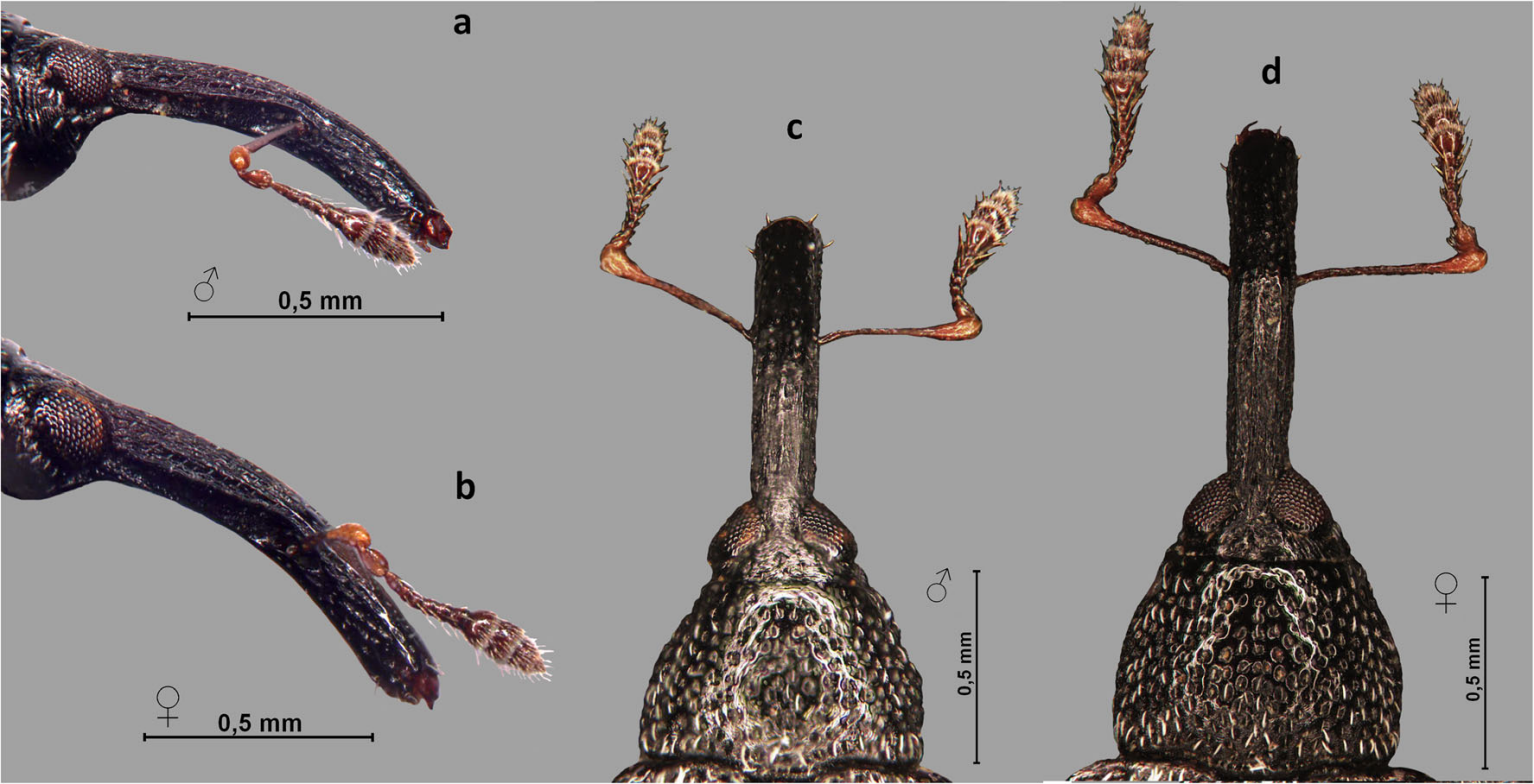
*Anthonomus santacruzi* Hustache, 1924, rostrum and pronotum: **a** lateral view of male; **b** lateral view of female; **c** dorsal view of male; **d** dorsal view of female (**a**–**d** optical microscope pictures).

**Fig 7:**
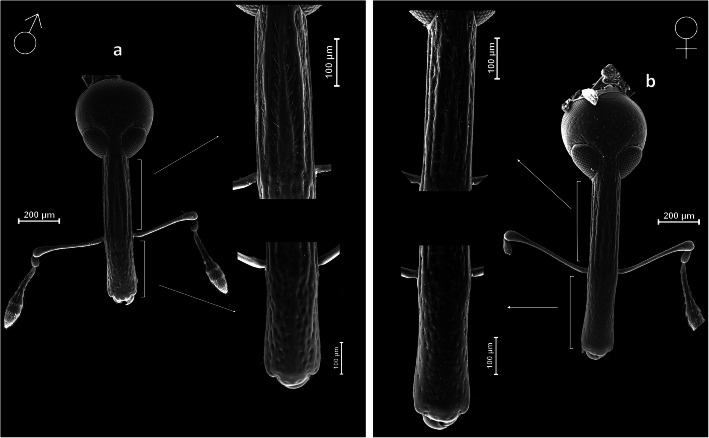
*Anthonomus santacruzi* Hustache, 1924, rostrum, dorsal view with magnification of the metarostrum and the apical part (SEM pictures): **a** male; **b** female.

The female rostrum length was significantly longer than the male rostrum in both, the total length (*t*_(95)_ = 7.416, *P* < 0.001) and length of metarostrum (*t*_(95)_ = 7.943, *P* < 0.001) (Table 1). The female metarostrum was almost half the length of the total rostrum length while for males it was only 40% of the total rostrum length (Table 1). On males, the surface of the metarostrum was distinctly ridged, with visible, longitudinal ridges, with the apical part densely covered by relatively coarse punctures, less shiny than the female. On females, the surface of metarostrum has fine, longitudinal ridges; apical part of rostrum glabrous, shiny, with sparse, shallow punctures, being relatively more smooth and shiny. Thus the female rostrum was found to be longer and smoother than the male rostrum, especially the apical part (Figs 5, 6a–d and 7a, b).

##### Fourth and fifth abdominal segments

**Fig 8:**
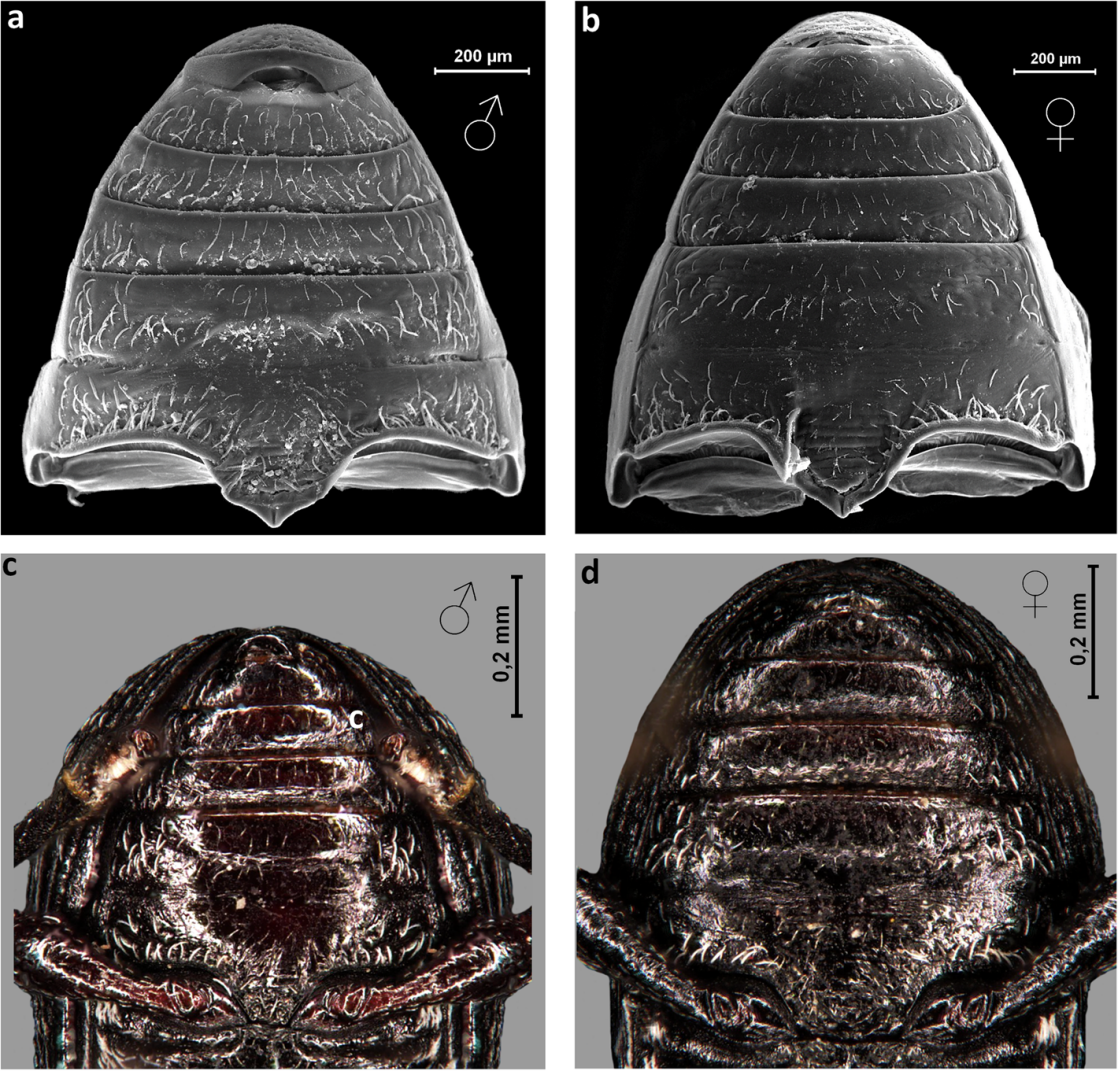
*Anthonomus santacruzi* Hustache, 1924, ventral view of abdominal segments: male (**a**, **c**), female (**b**, **d**) (**a**, **b** SEM pictures; **c**, **d** optical microscope pictures).

**Fig 9:**
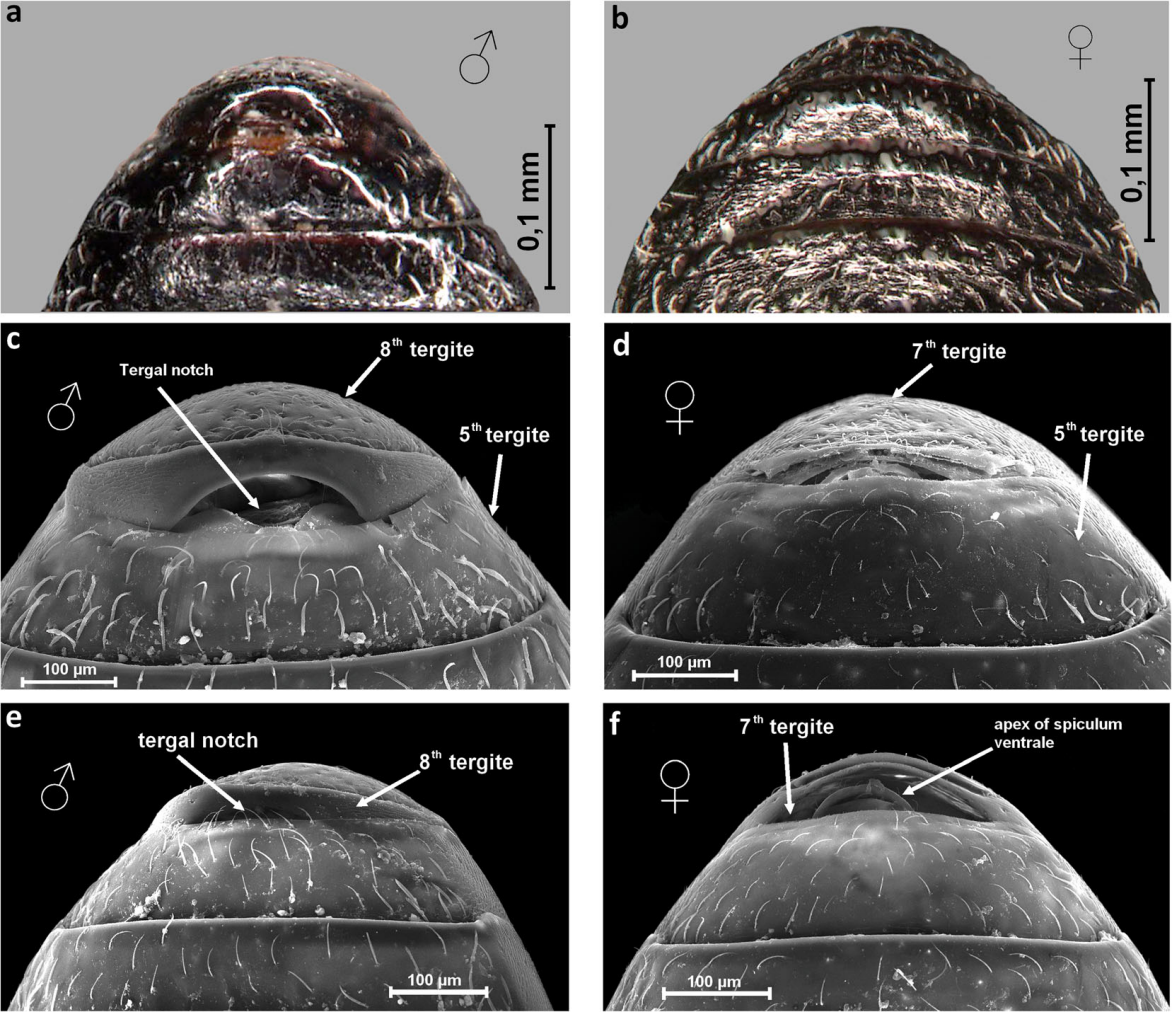
*Anthonomus santacruzi* Hustache, 1924, ventral view of abdominal segments: male (**a**, **c**, **e**), female (**b**, **d**, **f**) (**a**, **b** optical microscope pictures; **c**–**f** SEM pictures).

The 4th and 5th abdominal segments were clearly separated in males (Figs 8a, c and 9a, c, e), whilst they were fused together in females (Figs 8b, d and 9b, d, f). In addition, the male ventral side (1st–7th sternites) appears to have more hairs (Fig 8a), being slightly concave and shiny (Fig 8c), while the female ventral side (1st–7th sternites) appears to be less hairy (Fig 8b), flat or slightly convex and dull (Figs 8b, d).

##### Notch (7th and 8th tergite and sternite)

*Anthonomus santacruzi* males have eight visible dorsal abdominal segments, while females have seven visible segments. The notch, on the posterior edge of the 8th tergite was distinct in males (Table 1). The anterior border of the 7th tergite was thin and slightly rounded in females (Figs 9b, d, f). In contrast, the anterior border of the 8th tergite in males was distinctively augmented and notched (Figs 9a, c, e).

#### Other characters with sex-related differences

##### Pronotum shape and morphometrics

**Fig 10:**
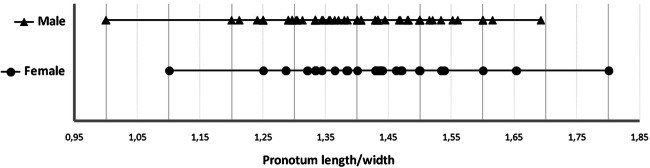
Length/width ratio of pronotum in male and female *Anthonomus santacruzi* Hustache, 1924.

On the lateral sides of the female pronotum (between the middle and base) it appears to be slightly curved, being “pear-shaped”, whereas in the males it is straight, being almost parallel. Pronotum length and width (Fig 10), showed no significant differences between the sexes in all specimens (*n* = 97) (length: *t*_(95)_ = 0.431, *P* = 0.668, width: *t*_(95)_ = 1.077, *P* = 0.284) (Table 1), but were still visible in some examined samples (Figs 2a, b and 6c, d).

##### Localisation of eyes

On females (in lateral view), the eyes do not stand out from the dorsal outline of the head, whereas in males (in lateral view) the eyes protrude slightly from the outline of the head (Figs 6a, b).

#### Selected characters without sex-related differences

**Fig 11:**
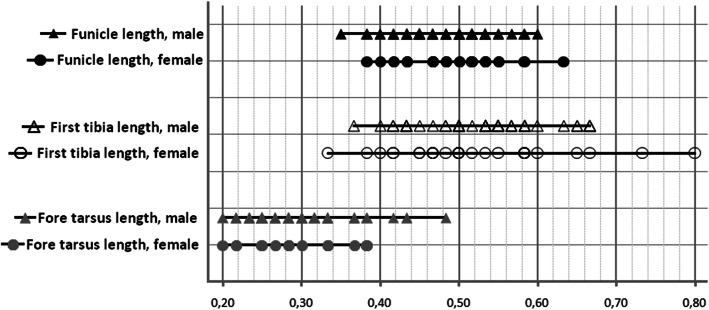
The range of variation in length of the funicle, first tibia and fore tarsus, between male and female *Anthonomus santacruzi* Hustache, 1924.

Funicule length (*t*_(95)_ = 1.089, *P* = 0.279), first tarsus length (*t*_(95)_ = 0.444, *P* = 0.658) and first tibia length (*t*_(95)_ = 0.202, *P* = 0.841) showed there were no significant differences between *A*. *santacruzi* sexes (Table 1; Fig 11).

## Discussion

Many researchers (e.g. Smreczyński 1972) reported distinct similarities between males and females of Anthonomini as well as rather less visible differences, e.g., relative length of rostrum (shorter on male, longer on female), the insertion of the scape (scape being situated closer to the apex of the rostrum in males than on females) and the sculpture of rostrum (distinctly ridged and matt in males and more smooth and shiny on females).

Even though total rostrum length (base–apex), length of metarostrum (antenna–apex), elytral length and width and total body length were significantly different between the sexes, they cannot be used to completely distinguish *A*. *santacruzi* males and females, as there is a broad overlap in these characters between the sexes. However, these disparities can be used for initial separation of sexes (George *et al* 2015).

Use of rostrum length was reported to be easy and has been widely used in distinguishing the sexes in selected *Anthonomus* species in the last century, but regarded as subjective because the characters were deemed subtle, variable and relative rather than absolute (Smreczyński 1972, Sappington & Spurgeon 2000).

However, the tergal notch was found to be the most useful body character in separating *A*. *santacruzi* sexes. All *A*. *santacruzi* males (*n* = 65) were successfully separated from all females (*n* = 32) using this notch. This supports Sappington & Spurgeon (2000) that if performed correctly, the notch method of separating males and females is 100% accurate. According to the article mentioned above, distinguishing sexes in insects using the notch is easy, to the extent that even a novice, who might mistake the 7th female tergite for the notch, will usually not make that mistake within the first hour of training in *A. grandis* sexing. The same, mentioned above authors, added that sexing using the notch is not as slow as generally perceived, and experienced personnel may process > 200–300 weevils in an hour.

Just like *A. grandis*, *A*. *santacruzi* males have eight dorsal abdominal segments while females have seven visible segments. However, this difference has not been used to distinguish sexes because it involves separating the elytra which the weevils keep tightly closed (Sappington & Spurgeon 2000).

Unfortunately, some of the body structure differences between the sexes, such as dorsal abdominal segments and genitalia, cannot be applied when dealing with live specimens as they can only be viewed using invasive procedures that may injure the specimens. Overall, the abdominal notch was found to be the most useful body structure that can be used to separate males and females in both live and preserved *A*. *santacruzi* weevils.

The rostrum of *A. santacruzi* females might have evolved into a longer and smoother structure to create oviposition places in the flower buds. Furthermore, the concave shape of the male weevil’s 1st–7th sternites might have evolved for mounting during mating compared with the female’s convex 1st–7th sternites.

The larger body size for females is a very widespread pattern in insects caused by the life history differences between females and males (Choe & Crespi 1997). According to Foelker and Hofstetter (2014), one of the potential explanations of this phenomenon is males and females often have different body sizes to avoid resource competition and enhance breeding or feeding efficiency (e.g. by utilizing different niches). In many groups of insects, females are larger than males also for this reason, and that females are filled with eggs.

Duan *et al* (1999) noted that different species in the genus *Anthonomus* can be sexually distinguished using different characters. For example, species such as *A. eugenii* and *A. texanus* Dietz, 1891 can be sexually distinguished using mucrones on the lateral side of the metatibian apex. This character does not exist in species such as *A. grandis*, *A. musculus* Say, *A. quadrigibbus* Say, 1831, *A. albopilosus* Dietz, 1891, *A. aeneolus* Dietz, 1891 and *A. pomorum*. Characters such as tarsal claws and shapes of ventral abdominal segments have also been used to distinguish sexes in *A. grandis*, *A. eugenii*, *A. signatus* and other anthonomine weevils. But, finally, it appears that species such as *A. grandis*, *A*. *pomorum* and *A*. *santacruzi* can be better sexually distinguished using the differences in the last abdominal segments (Duan *et al* 1999; Sappington & Spurgeon 2000).

Taking into consideration all the above-mentioned observations of sex-related characters on *A*. *santacruzi*, these can be divided into two groups:

1. The “main characters”, clearly visible on most of the studied specimens (*n* = 97) (features marked in italic in Table 1), were rostrum length, elytra length, presence of notch and total body length. Some of these characters are related to the structure of the cuticle (especially of the rostrum) and shape of some body parts (rostrum, abdominal sternites) or arise from different proportions of the body parts.
2. There were also some “subsidiary characters”. We add here other features such as the shape of the pronotum and the dorsal localisation of eyes, which due to individual variability sometimes make them difficult to use to distinguish between sexes.

Taking into consideration other groups of Curculionidae, Baba & Yoneda (2000) observed that differences between the sexes depended on the density and shape of scales on the metasternum of the West Indian sweetpotato weevil—*Euscepes postfasciatus* Fairmaire, 1849, while Grocholski *et al* (1976) observed sex-related differences in patterns of bristles on the last abdominal sternite of bark beetles in the genus *Hylastes* (Erichson, 1836). Further, the presence of an elongated depression on the abdominal sternites of males was noted on *Hylobius radicis* (Buchanan, 1935) by Wilson (1966) and on *H. warreni* (Wood, 1957) by Öhrn *et al* (2008), as well on the cocoa weevil borer—*Pantorhytes szentivanyi* (Marshall, 1957) by Hassan (1973). Consequently, Santos *et al* (2018) reported differences in the shape of the abdominal tergites between males and females of *Ozopherus muricatus* Pascoe, 1872. Hence, external dimorphism in weevils shows fairly large variability across species, and thus, further study on this topic is required.

The male-biased sex ratio of 0.67 (one female (*n* = 32) to two (*n* = 65) males) in the sampled weevils used in this study might be a result of protandry (i.e. early male emergence). The biased sex ratio has been found to be promoted by climate alterations in the timing of rainfall and/or extreme temperatures (e.g. the Mediterranean acorn weevil *Curculio elephas* (Gyllenhal, 1836) (Coleoptera: Curculionidae) (Bonal *et al* 2015). Protandrous behaviour and sex ratios in general have not previously been reported in the genus *Anthonomus* (except for sex ratios in *A. grandis*, e.g. Greenberg *et al* 2004) and may warrant a detailed investigation. Protandry has the advantage of preserving the effective population size as the females will not be male limited, and unlike a female-biased sex ratio, it will not be detrimental to population genetic diversity. However, strongly biased adult sex ratios influence the risk of extinction or population collapse (Greenberg *et al* 2004). Hence, the optimal sex ratios of insects released in biocontrol programs to maximize their effectiveness need more research in general.

## Electronic Supplementary Material

ESM 1 (XLSX 17 kb)
